# Hsa_circ_0008434 regulates USP9X expression by sponging miR-6838-5p to promote gastric cancer growth, migration and invasion

**DOI:** 10.1186/s12885-021-09052-4

**Published:** 2021-12-02

**Authors:** Xin Xu, Shoulian Wang, Haibo Wang, Chunpeng Pan, Wenyan Yang, Jiwei Yu

**Affiliations:** 1 Department of General Surgery, Shanghai Ninth People’s Hospital, School of Medicine, Shanghai Jiao Tong University, 639 Zhizaoju Road, Shanghai, 200011 China; 2 Department of Gastroenterology, Shanghai Ninth People’s Hospital, School of Medicine, Shanghai Jiao Tong University, 639 Zhizaoju Road, Shanghai, 200011 China

**Keywords:** hsa_circ_0008434, Gastric cancer, Proliferation, Migration, Invasion

## Abstract

**Background:**

The role of circular RNAs (circRNAs) in the occurrence and development of gastric cancer (GC) has recently attracted increasing interest. The following study investigates the role of a newly discovered hsa_circ_0008434, which has been confirmed to be highly expressed in GC tissues, in regulating GC biological behaviour.

**Methods:**

High-throughput RNA sequencing was used to identify differentially expressed genes between normal gastric tissues and GC tissues; actinomycin D and RNase R assays were used to determine the stability and loop structure of hsa_circ_0008434; and the miRanda database was used to predict the target genes of hsa_circ_0008434. The role of hsa_circ_0008434 in cell proliferation, migration, and invasion was examined using CCK-8, wound healing, Transwell and colony formation assays. The regulatory relationships among hsa_circ_0008434, microRNA-6838 (miR-6838), and ubiquitin-specific peptidase 9X (USP9X) were determined by dual-luciferase activity assays. The expression of hsa_circ_0008434 and miR-6838 was measured by qPCR; the expression of USP9X was detected by immunohistochemistry and Western blotting. The effects of hsa_circ_0008434 on in vivo tumour growth were assessed in xenograft models.

**Results:**

We found that hsa_circ_0008434 was one of the most upregulated circRNAs in GC tissue versus normal tissue. Further in vitro testing indicated that by acting as a miRNA sponge for miR-6838-5p, hsa_circ_0008434 promotes the expression of USP9X and further increases the proliferation, migration, and invasion of GC cells. In addition, animal studies indicated that hsa_circ_0008434 could promote tumour growth in vivo.

**Conclusions:**

Hsa_circ_0008434 may promote GC proliferation, invasion and migration by regulating the expression of miR-6838 and USP9X.

**Supplementary Information:**

The online version contains supplementary material available at 10.1186/s12885-021-09052-4.

## Background

Gastric cancer (GC) is the third leading cause of cancer-related death worldwide [1]. GC is one of the most frequently occurring cancers in China, with an estimated 60,000 new cases occurring each year [2]; stage IV GC accounts for approximately 10% ~ 20% of GC cases [3].

Circular RNAs (circRNAs) are a class of endogenous noncoding RNAs with no 3′-cap structure and a 5′-poly A tail [4], and these ends covalently connect to form a closed ring structure that is not degraded by ribonucleic acid exonuclease. CircRNAs regulate gene and protein expression through a variety of mechanisms, including the regulation of transcription and selective splicing, interaction with RNA-binding proteins (RBPs), and adsorption of functional microRNAs (miRNAs), thereby eliminating the inhibitory effect of miRNAs on their target genes and increasing the expression level of target genes [5]. Previous studies have found that circRNAs have an important role in the occurrence and development of cancer [6]. Nevertheless, since most of the ring structures have not yet been discovered, further studies are needed to elucidate their functions.

The role of circRNAs in the occurrence and development of GC has recently gained increasing research interest. In 2015, Li et al. found that hsa_circ_002059 was downregulated in GC [7]. Moreover, Zhang et al. found that CIRC NRIP1 can promote the progression of GC [8] by inhibiting the expression of miRNA-149-5p. These findings suggest that circRNAs are closely related to the initiation and progression of GC. In this study, high-throughput sequencing of GC tissues and adjacent tissues revealed a significant increase in hsa_circ_0008434 expression in GC tissues.

MiRNAs are noncoding single-stranded small RNAs [9] with a length of approximately 22 nucleotides in organisms. As a kind of noncoding small molecule RNA, it regulates the transcription and expression of 30–50% of genes, regulates various cellular pathways, including autophagy, and takes miRNAs as a target as a new cancer treatment strategy [10]. Ubiquitin-specific peptidase 9X (USP9X) is a ubiquitin protease that has recently been proven to play an important role in cancer development [11]. Recent studies have shown that USP9X can regulate many biological processes, including cell polarization, autophagy, apoptosis, protein transport, cell growth, migration and stem cell maintenance [12–14]. We found that USP9X was the target gene of miR-6838 through bioinformatics analysis and MIRDB website (http://mirdb.org/) prediction.

In this study, we examined the role of a newly discovered hsa_circ_0008434, which has been confirmed to be highly expressed in GC tissues, in regulating GC biological behaviour in vitro and in vivo.

## Methods

### Clinical samples

A total of 13 paired tumour samples and adjacent healthy tissue samples were collected from GC patients who received surgical treatment in 2018 at Shanghai Ninth People’s Hospital. All 13 GC patients included in this study were pathologically diagnosed with moderately to poorly differentiated adenocarcinomas, with pathological stage III, and all required conventional chemotherapy after surgery. Written consent was obtained from all patients involved, and the study was approved by the Institutional Ethical Review Board of Shanghai Ninth People’s Hospital, School of Medicine, Shanghai Jiao Tong University.

### Cell culture and transfection

Normal human gastric epithelial cell lines GES-1 and GC (SGC7901, MKN-45, AGS) were obtained from the cell bank of Fudan University. Cells were cultured in DMEM supplemented (Gibco, Grand Island, NY, USA) with 10% heat-inactivated fetal bovine serum (FBS) (Gibco, Grand Island, NY, USA), 100 U/ml penicillin and 100 μg/ml streptomycin (Gibco, Grand Island, NY, USA) in a humidified atmosphere containing 5% CO_2_/95% air at 37 °C.

SGC7901 cells were transfected with three hsa_circ_0008434 siRNA fragments, one USP9X siRNA fragment and one antagomiR-6838 fragment using Lipofectamine 3000 (Invitrogen, Carlsbad, CA, USA), according to the manufacturer’s instructions. The hsa_circ_0008434 siRNAs and antagomiR-6838 sequences are shown in Table 1. All siRNAs and antagomiRs sequences were provided by GenePharma (Shanghai, China).

**Table 1:** The sequences of hsa_circ_0008434, USP9X siRNAs and antago-miR-6838

siRNA	Sequences
si-#1	GCACCAGCCUGCAUCUAUUTT AAUAGAUGCAGGCUGGUGCTT
si-#2	CCUAAAUGCUUCUUCACUUTT AAGUGAAGAAGCAUUUAGGTT
si-#3	GCAGGAAGACUCUUAUUUATT UAAAUAAGAGUCUUCCUGCTT
antagomiR-6838	CGGAAGUCCUGCUUCUGUUGC AACAGAAGCAGGACUUCCGUG
si-USP9X	GAAAUAACUUCCUACCGAA

### High-throughput RNA sequencing

TRIzol reagent was used to isolate total RNA from three groups of GC tissues and paired normal tissues. After RNA quality inspection and rRNA depletion, sequencing was completed by the Shenzhen HaploX Genomics Center, and the sequencing platform was Illumina PE150.

Find_circ software was used to identify circRNAs. Based on the prediction and screening results for circRNAs, the length distribution of circRNA in each sample was recorded and plotted. The number of circRNAs located on each chromosome in all samples was counted to show the distribution of circRNAs on different chromosomes. The transcript per million (TPM) values of reads count of circRNA in each sample were converted. In the transcriptome analysis, principal component analysis (PCA) reduced the large amount of gene expression information for the samples to a few independent principal components for comparison between samples.

Cluster analysis was used to classify samples and circRNAs. The similarities in circRNA expression patterns among different samples can be demonstrated via clustering diagrams of different circRNAs. The gene ontology (GO) and Kyoto Encyclopedia of Genes and Genomes (KEGG) functional enrichment of differentially circRNA-derived genes were conducted to explore the role of circRNAs and their host genes.

### Q-PCR

Total RNA was extracted from GC cells with TRIzol reagent (Invitrogen, Carlsbad, CA, USA) following the manufacturer’s protocol. The concentration of the isolated RNA was measured and transcribed to obtain complementary cDNA using the RevertAidTM First Strand cDNA Synthesis Kit (Takara Bio, Shiga, Japan).

According to the instructions of Invitrogen’s RNA extraction kit, RNA content determination was measured. And a reverse transcription kit was used to reverse transcribe the secretory RNA into cDNA. Using a 20 μl reaction system for PCR amplification and detection by BIO-RAD fluorescent real-time PCR. Gene-specific primer sequences are shown in Table 2, and the primers were provided by Sangon Biotech (Shanghai, China).

**Table 2:** The sequences of gene primers

Gene Primers	Sequences
hsa_circ_0008434-Forward	5′-AGCAACACCACCAGCCATC-3′
hsa_circ_0008434-Reverse	5′-GACTCTGTCATACTTTACCCATTTC-3’
UQCRC2-Forward	5′-AGCAACACCACCAGCCATC-3’
UQCRC2-Reverse	5′-TAAATCCCAAAGAGTCCAG-3’
miR-6838-Forward	5′-GCACTCCTGGATGCCAATCT-3’
miR-6838-Reverse	5′-CTCTACAGCTATATTGCCAGCCAC-3’
USP9X-Forward	5′-TCGGAGGGAATGACAACCAG-3’
USP9X-Reverse	5′-GGAGTTGCCGGGGAATTTTCA-3’
GAPDH-Forward	5′-GGTCGGAGTCAACGGATTTG-3’
GAPDH-Reverse	5′-ATGAGCCCCAGCCTTCTCCAT-3’

### Western blotting analysis

The cells were lysed with radioimmunoprecipitation assay lysis buffer (RIPA, Beyotime, Shanghai, China) mixed with aphenylmethanesulfonyl fluoride protease inhibitor at a 100:1 ratio (Beyotime, Shanghai, China). The protein concentrations were determined using a Bicinchoninic Acid Protein Assay Kit (Beyotime, Shanghai, China).

A 30 μg protein sample was used for 10% sodium dodecyl sulfate polyacrylamide gel electrophoresis. After electrophoresis, the protein sample was transferred to PVDF membrane (Millipore) by the wet conversion method, and the skimmed milk powder was sealed. After overnight incubation with primary antibodies at 4 °C, TBS buffer was added to wash the membrane, and then incubated at room temperature for 2 h with secondary antibody (Abcam). TBST buffer was used for washing, and ECL reagent (Beyotime, Shanghai, China) was used for band visualization. The expression of the target protein was equal to the greyscale value of the target band/the greyscale value of the GAPDH band. The primary antibodies included anti-USP9X (Abcam, ab19879) and anti-GAPDH (Zhongshan Jinqiao, TA-08).

### Histology and immunohistochemistry

For these experiments, we followed the steps provided by Abcam. GC tissues and paired normal tissues were incubated with an antibody against USP9X (Abcam) at 4 °C overnight. Then, they were incubated with secondary antibody for 2 h at room temperature.

### Detection of dual-luciferase activity

The dual-luciferase reporter assay was performed by using the Dual Luciferase Assay System Kit (Promega, Madison, WI, USA) to verify whether hsa_circ_0008434 and USP9X were direct target genes of miR-6838, according to the manufacturer’s instructions. The hsa_circ_0008434 and USP9X 3′ untranslated regions (UTRs) were synthesized and cloned into pmirGLO luciferase reporter vectors (Promega) to generate luciferase reporter plasmids. Two hundred ninety-three T cells (1 × 10^5^ cells/well) were cotransfected with luciferase reporter plasmids and the miR-6838 mimics and miR-6838 mimics inhibitor (50 nmol/L) using Lipofectamine 3000 (Invitrogen). After 48 h of transfection, luciferase activity was measured using a Dual-Luciferase Reporter Assay System (Promega). The relative luciferase activity was determined by the ratio of Renilla luciferase activity to firefly luciferase activity. The experiment was repeated three times.

### Cell Counting Kit-8 (CCK-8) assay

GC cells were transfected with the corresponding oligonucleotides (si-hsa_circ_0008434 and si-NC). The GC cells at a concentration of 4 × 10^3^ cells/ml were inoculated in 96 well plates and cultured at 37 °C for 96 h. CCK-8 solution (10 μl, Beyotime, Shanghai, China) was added to 100 μl medium containing 10% FBS every 24 h. The absorbance values were measured at 450 nm.

### Wound healing assay

Cells were seeded into 12-well culture plates at a density of 1 × 10^5^ cells/well and cultured for 12 h using serum-free medium. After scraping the cells with a pipette tip, the detached cells were washed away with medium, and the remaining cells were photographed at 0 and 24 h.

### Transwell assay

Transwell chambers (8-μm pores, Corning Incorporated, USA) with Matrigel matrix (BD Biosciences, USA) were used to examine the invasion ability of GC cells. In brief, treated GC cells were resuspended in serum-free medium at a final concentration of 1 × 10^5^ cells/ml. In addition, 200 μl suspended GC cells were added to the upper chamber, and 500 μl medium with 10% FBS was added to the lower chamber. After 24 h of incubation at 37 °C with 5% CO_2_, the invaded GC cells were fixed in methanol and stained with 0.5% crystal violet (Beyotime, Shanghai, China).

### Colony formation assay

For the colony formation assay, GC cells were seeded in 6-well plates at a density of 1 × 10^3^/well and cultured at 37 °C for 14 days. The colonies were stained with 0.1% crystal violet and 20% methanol solution. The cells were then counted and analysed.

### RNA-fish

Digoxin-labelled probes specific to hsa_circ_0008434 (DIG-UTP, Roche, 11,209,256,910) and biotin-labelled probes against to miR-6838 (Biotin RNA Labelling Mix, Roche, 11,685,597,910) were prepared by Servicebio Wuhan. The probe sequences for hsa_circ_0008434 and miR-6838 were 5′-TTTACCCATTTCCTTGGCAGCTTGGACATC-3′ and 5′-AGGAGTCTTGCCACTGCTGCTT-3′, respectively. Hybridization was performed overnight with 1 μM hsa_circ_0008434 probe and 1 μM miR-6838-5p probe. The signals were detected by Cy3-conjugated anti-digoxin (0.01 mg/ml) and FITC-conjugated anti-biotin (0.02 mg/ml) antibodies (Jackson ImmunoResearch Inc., West Grove, PA, USA). The cell nuclei were counterstained with 4,6-diamidino-2-phenylindole (DAPI). Finally, the images were analysed on a Nikon Eclipse CI.

### Actinomycin D assay

SGC7901 and MKN45 cells were exposed to actinomycin D (2 μg/ml, Sigma) to block transcription for 4, 8, 12, and 24 h. Then, the cells were collected, and the stability of hsa_circ_0008434 and UQCRC2 mRNA was analysed by q-PCR.

### RNase R treatment

Total RNA (2 μg) was incubated for 20 min with or without 3 U/μg at 37 °C. The resulting RNA was purified using the RNeasy MinElute Cleanup Kit (Qiagen).

### Tumour xenograft model

BALB/c male nude mice, 4 weeks old, weighing 20–25 g, were obtained from Shanghai Jie Si Jie Laboratory Animal Co., Ltd., China. All animals were housed in an environment with a temperature of 22 ± 1 °C, relative humidity of 50 ± 1%, and a light/dark cycle of 12/12 h.

Mice were randomly divided into two groups: the hsa_circ_0008434 knockdown group and the si-NC group. The hsa_circ_0008434 knockdown group received and subcutaneous injection of SGC7901 cells (1 × 10^7^) with hsa_circ_0008434-knockdown, while the si-NC group received the same amounts of cells as the si-NC group. Mice were euthanized 3 weeks after injection, after which tumour tissue was collected and further analysed in ex vivo.

### Statistical analyses

SPSS 25.0 (IBM, Chicago, IL, USA) was used to analyse the statistical data. The results are shown as the mean ± SD. Chi-square tests, Student’s t-tests, and analysis of variance (ANOVA) were used to assess the statistical significance of differences between two or more groups. Pearson correlation analysis was used to analyse the correlations. A *P* value of < 0.05 was considered statistically significant.

## Results

### Hsa_circ_0008434 is expressed at a high level in GC and maintains a highly stable loop structure

**Fig. 1:**
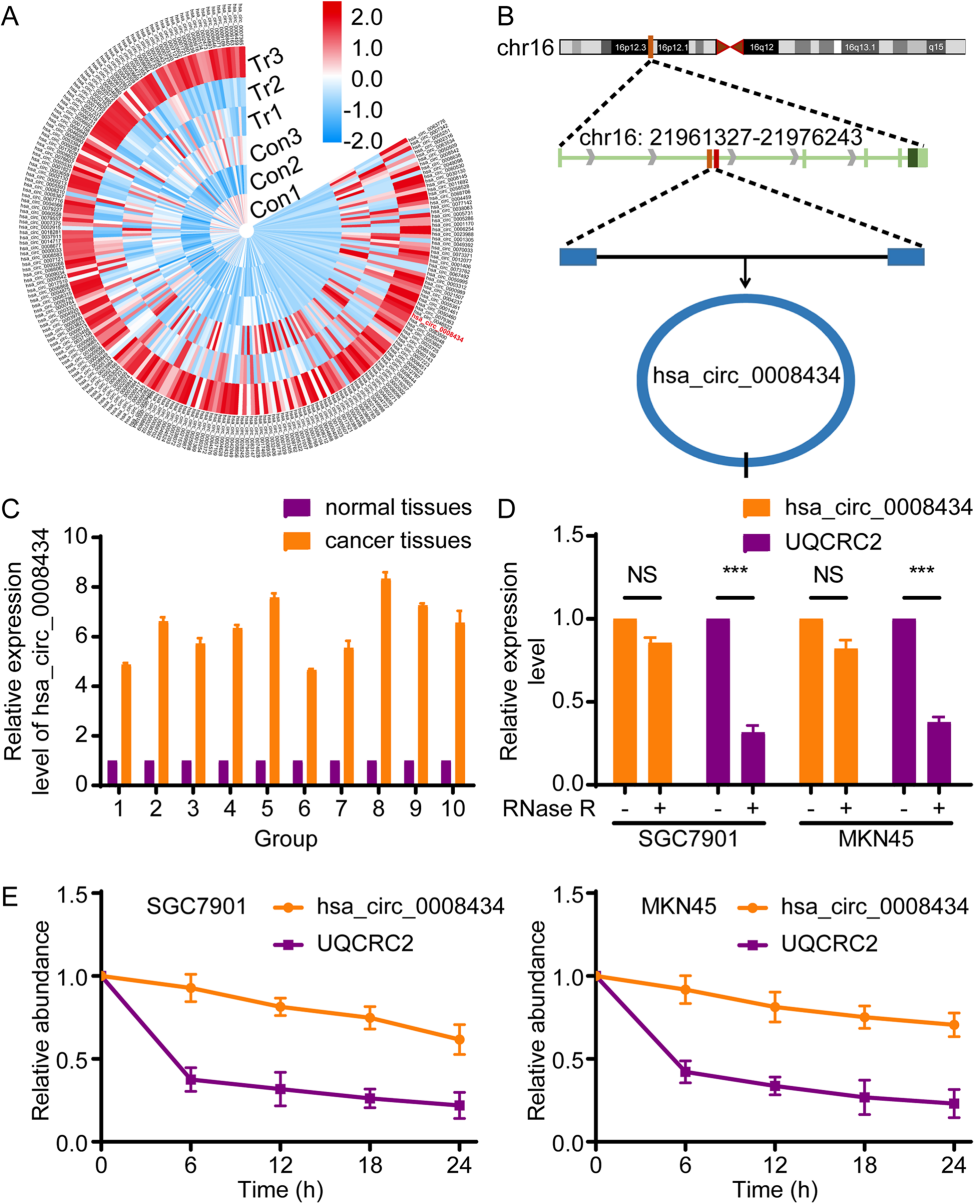
Hsa_circ_0008434 expression is upregulated in GC tissues. **A** Heatmap of the top differentially expressed circRNAs in three paired human GC tissues and matched nontumour tissues. Red colour and blue colour indicate high and low expression, respectively. **B** The genomic loci of the UQCRC2 gene and hsa_circ_0008434. **C** The relative mRNA expression level of hsa_circ_0008434 in 10 paired human GC tissues and matched nontumour tissues. **D** qRT-PCR analysis results of hsa_circ_0008434 and UQCRC2 mRNA after treatment with RNase R in SGC7901 and MKN45 cells. **E** qRT-PCR analysis of the abundances of hsa_circ_0008434 and UQCRC2 in SGC7901 and MKN45 cells after treatment with actinomycin D at the indicated time points. ^***^p < 0.001; NS, no significance

To determine the involvement of circRNAs in the progression of GC, we explored the expression profile of circRNAs in GC tissues by using high-throughput RNA sequencing (RNA-seq). CircRNA analysis was performed on tumour tissues and paired normal gastric tissues that were collected from three GC patients. In total, 576 circRNAs were identified as differentially expressed in GC tissues versus normal tissues, and hierarchical clustering analysis revealed that hsa_circ_0008434 was one of the most upregulated circRNAs in GC tissue (Fig. 1A). Then, we performed GO and KEGG pathways enrichment analyses on these 576 circRNAs and host genes, and found that hsa_circ_0008434 plays an important role in the progress of GC (see supplementary materials). Consequently, hsa_circ_0008434 was selected for further examination.

In the RNA-seq results, according to the chromosome distribution, start/stop sites and length of circRNAs, we found that hsa_circ_0008434 is located on chromosome 16p12.2 and transcribed from the host gene UQCRC2 (Fig. 1B). We further assessed the expression level of hsa_circ_0008434 in ten other GC tissues and paired normal gastric tissues by using q-PCR. Our results revealed that the expression of hsa_circ_0008434 was higher in GC tissues than in normal gastric tissues (Fig. 1C).

Next, we treated GC cell lines (SGC7901 and MKN45) with RNase R, and a lower digestion rate indicated better resistance of hsa_circ_0008434 and confirmed the loop structure of hsa_circ_0008434 (Fig. 1D). Moreover, we treated the two cell lines with the transcription inhibitor actinomycin D, and the half-life of hsa_circ_0008434 reached 24 h (6.5 h for linear UQCRC2) (Fig. 1E), indicating that hsa_circ_0008434 has better stability.

### Silencing hsa_circ_0008434 can inhibit the proliferation, invasion and migration of GC cells

**Fig. 2:**
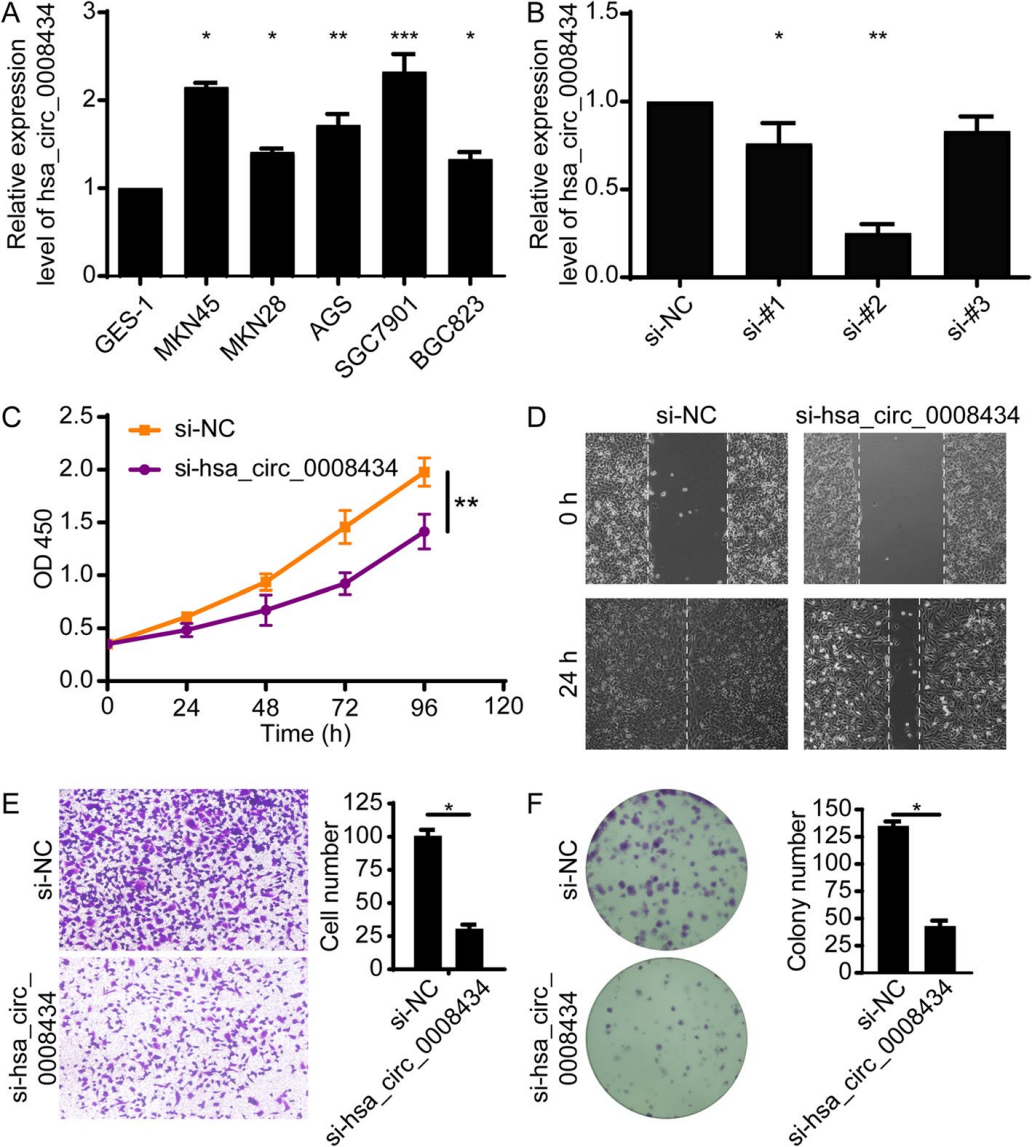
Hsa_circ_0008434 promotes GC cell proliferation, migration and invasion in vitro. **A** qRT-PCR was performed to identify the relative mRNA expression level of hsa_circ_0008434 in GES-1, MKN45, MKN28, AGS, SGC7901, and BGC823 cells. **B** qRT-PCR analysis confirmed the transfection effects of the three siRNAs. **C** A CCK-8 assay was used to verify the proliferation ability of SGC7901 cells with or without hsa_circ_0008434 knockdown. **D** A wound healing assay was conducted to explore the migration ability of SGC7901 cells with or without hsa_circ_0008434 knockdown. **E** Transwell assays were performed to examine the invasion ability of SGC7901 cells with or without hsa_circ_0008434 knockdown. **F** A colony formation assay was performed to determine the colony formation ability of SGC7901 cells with or without hsa_circ_0008434 knockdown. ^*^p < 0.05; ^**^p < 0.01; ^***^p < 0.001

Next, we examined whether hsa_circ_0008434 could affect the biological behaviour of GC. The expression of hsa_circ_0008434 was first analysed and compared in five GC cell lines (MKN45, MKN28, AGS, SGC7901, BGC823) and normal human gastric epithelial cell line (GES-1) using q-PCR (Fig. 2A). The highest expression of hsa_circ_0008434 was found in SGC7901 cells, which were selected for further in vitro testing.

Hsa_circ_0008434 in SGC7901 cells was then silenced using three siRNA fragments, and si-#2 exhibited the best efficiency, as determined by qPCR, and was chosen for the knockdown experiments (Fig. 2B). A lower proliferation rate was found in cells treated with si-#2 compared to control cells (Fig. 2C). In addition, the wound healing assay and Transwell assay indicated that the downregulation of hsa_circ_0008434 significantly suppressed cell migration and invasion (Fig. 2D and E). The colony formation assay showed that inhibition of hsa_circ_0008434 expression could significantly reduce the colony formation ability of GC cells (Fig. 2F). These data further confirmed that hsa_circ_0008434 plays an important role in the proliferation, invasion and migration of GC cells.

### Hsa_circ_0008434 acts as a miRNA sponge for miR-6838-5p

**Fig. 3:**
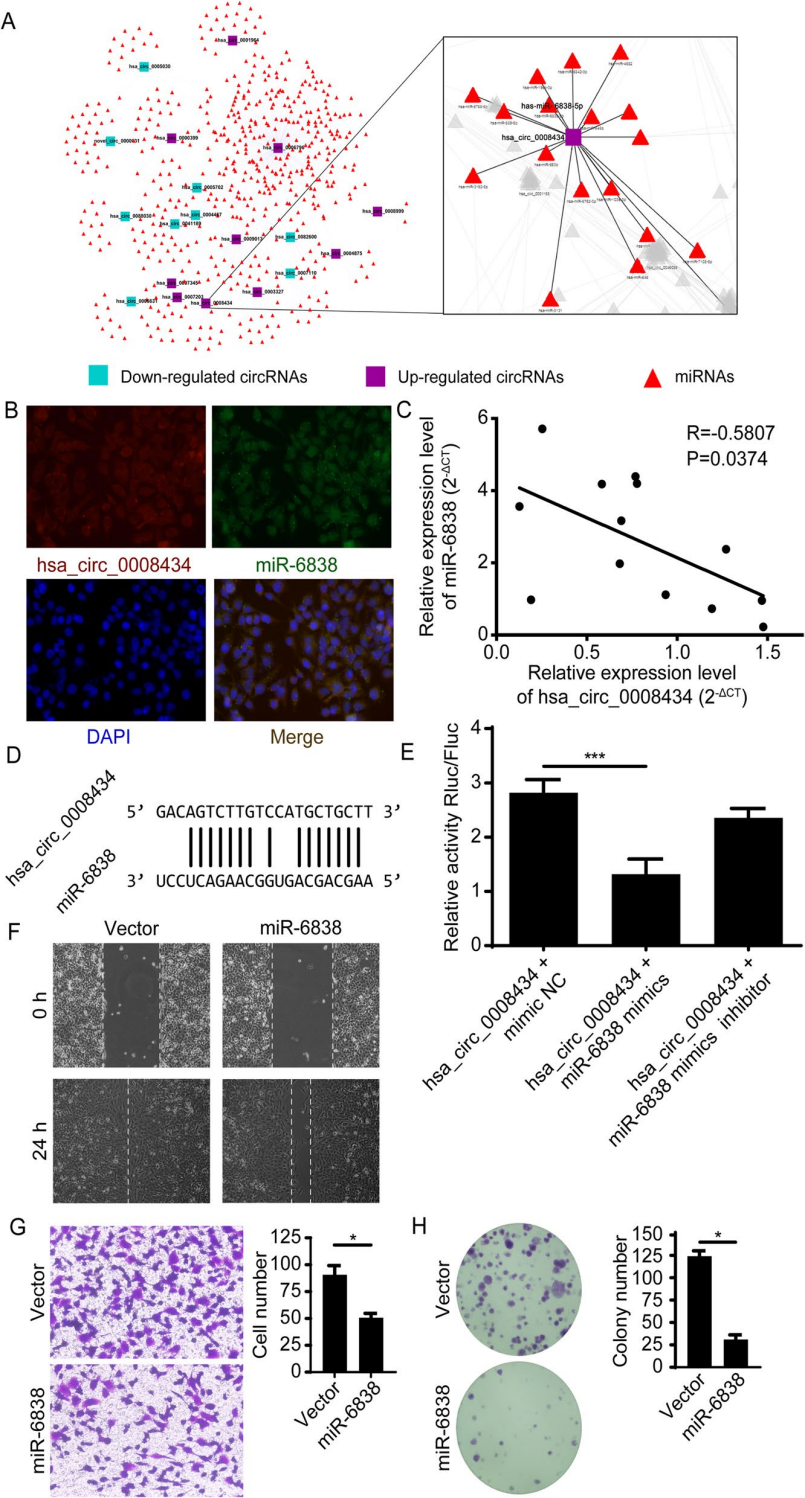
Hsa_circ_0008434 serves as a miRNA sponge for miR-6838-5p. **A** A miRNA-circRNA-gene regulatory network was established via the miRanda database. **B** RNA-FISH was carried out to assess the colocalization of hsa_circ_0008434 and miR-6838-5p. Blue represents DAPI nuclear staining, red represents hsa_circ_0008434 staining and green represents miR-6838 staining. **C** Pearson correlation analysis was used to determine the correlation of hsa_circ_0008434 expression with miR-6838-5p in GC tissues. **D** The potential binding sites between hsa_circ_0008434 and miR-6838-5p were determined via the MiRanda database. **E** A dual-luciferase reporter assay was performed in HEK-293 T cells, and hsa_circ_0008434 cotransfection with miR-6838-5p mimics led to decreased luciferase activity. **F** A wound healing assay was conducted to explore the migration ability of SGC7901 cells with or without miR-6838 upregulation. **G** Transwell assays were performed to examine the invasion ability of SGC7901 cells with or without miR-6838 upregulation. **H** A colony formation assay was performed to assess the colony formation ability of SGC7901 cells with or without miR-6838 upregulation. ^*^p < 0.05; ^**^p < 0.01; NS, no significance

It is well known that circRNAs can act as miRNA sponges to regulate miRNA targets [15]. Previous studies indicated that miR-6838-5p acts as a tumour suppressor in triple-negative breast cancer [16] and may also promote the development of renal cell carcinoma [17]. In this study, we examined the relationship between hsa_circ_0008434 and miR-6838. After predicting the binding sites of hsa_circ_0008434 and analysing the regulatory network of the miRNA-circRNA, we explored the potential interactions between hsa_circ_0008434 and miR-6838 (Fig. 3A). Then, we performed RNA-FISH and found that hsa_circ_0008434 and miR-6838 colocalized in the cytoplasm of GC cells (Fig. 3B). Furthermore, we observed a negative correlation between miR-6838 and hsa_circ_0008434 in GC tissues (Fig. 3C).

To explore whether there is a regulatory relationship between hsa_circ_0008434 and miR-6838, we first evaluated the potential binding sites between hsa_circ_0008434 and miR-6838 in gene sequences using the miRanda database (Fig. 3D). Through the dual-luciferase activity assay, we found that miR-6838-5p mimics weakened the fluorescence signal in 293 T cells transfected with hsa_circ_0008434 but not in cells transfected with a miR-6838-5p mimic inhibitor (Fig. 3E).

To determine whether miR-6838 has an effect on the adverse biological behaviour of GC cells, wound healing assays and Transwell assays were carried out and indicated that the upregulation of miR-6838 could significantly inhibit cell migration and invasion (Fig. 3F and G). The colony formation assay showed that overexpression of miR-6838 could significantly decrease the colony formation ability of GC cells (Fig. 3H). All of the above data proved that miR-6838 plays a protective role in the proliferation, invasion and migration of GC cells.

### Hsa_circ_0008434 regulates the expression of USP9X via miR-6838

**Fig. 4:**
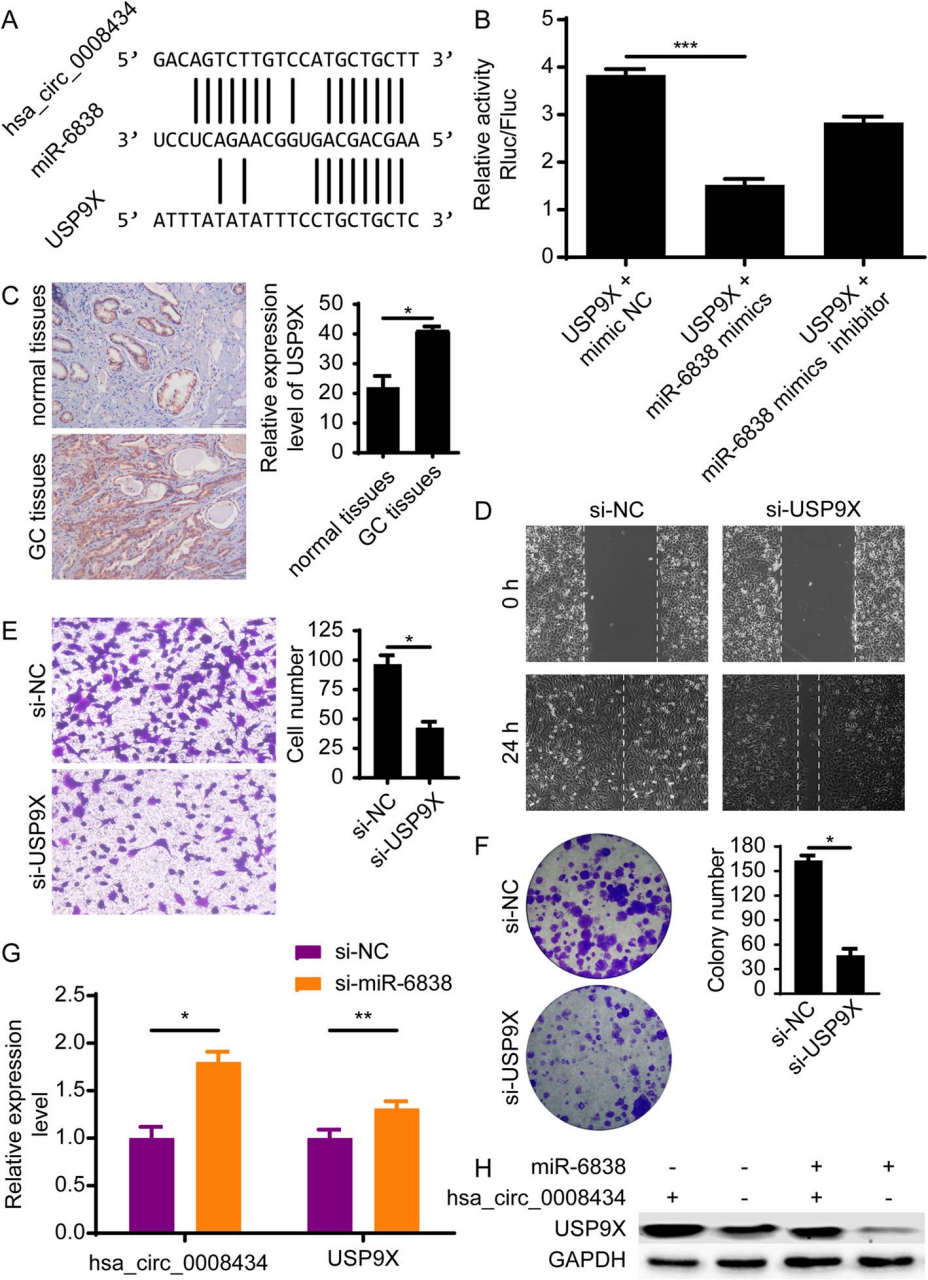
Hsa_circ_0008434 regulates USP9X expression via miR-6838. **A** The potential binding sites between USP9X and miR-6838-5p were predicted with the miRanda database and MiRDB website. **B** Luciferase reporter assays showed that USP9X cotransfection with miR-6838-5p mimics led to decreased luciferase activity. **C** The relative expression of USP9X was examined by immunohistochemical staining analysis. **D** A wound healing assay was conducted to explore the migration ability of SGC7901 cells with or without USP9X knockdown. **E** Transwell assays were performed to examine the invasion ability of SGC7901 cells with or without USP9X knockdown. **F** A colony formation assay was performed to identify the colony formation ability of SGC7901 cells with or without USP9X knockdown. **G** qRT-PCR was performed to identify miR-6838’s negative regulation of USP9X by hsa_circ_0008434. **H** Western blot analysis was performed to prove that hsa_circ_0008434 regulates USP9X expression via miR-6838. ^*^p < 0.05; ^**^p < 0.01; NS, no significance

miRNAs usually act as gene regulators by inhibiting the expression of their paired target mRNAs [18]. According to the miRanda database and MiRDB website (http://mirdb.org/), USP9X may serve as a potential target gene of miR-6838 (Fig. 4A). This was further proven by a dual-luciferase activity assay, as the fluorescence signal in 293 T cells transfected with USP9X was weakened by miR-6838 mimics (Fig. 4B). Furthermore, immunohistochemical staining showed that the expression of USP9X was significantly higher in GC tissues than in normal tissues (Fig. 4C). The wound healing assay and Transwell assay revealed that the downregulation of USP9X significantly suppressed cell migration and invasion (Fig. 4D and E). The colony formation assay showed that the inhibition of USP9X expression could significantly reduce the colony formation ability of GC cells (Fig. 4F). Moreover, after silencing miR-6838, the expression of hsa_circ_0008434 and USP9X was upregulated, which proved the negative regulatory effect of miR-6838 on these two genes (Fig. 4G). The rescue experiment showed that miR-6838 was able to weaken the upregulation of USP9X induced by hsa_circ_0008434 (Fig. 4H). The above data proved our hypothesis that hsa_circ_0008434 regulates the expression of USP9X via miR-6838.

### Hsa_circ_0008434 promotes the growth of tumours in vivo

**Fig. 5:**
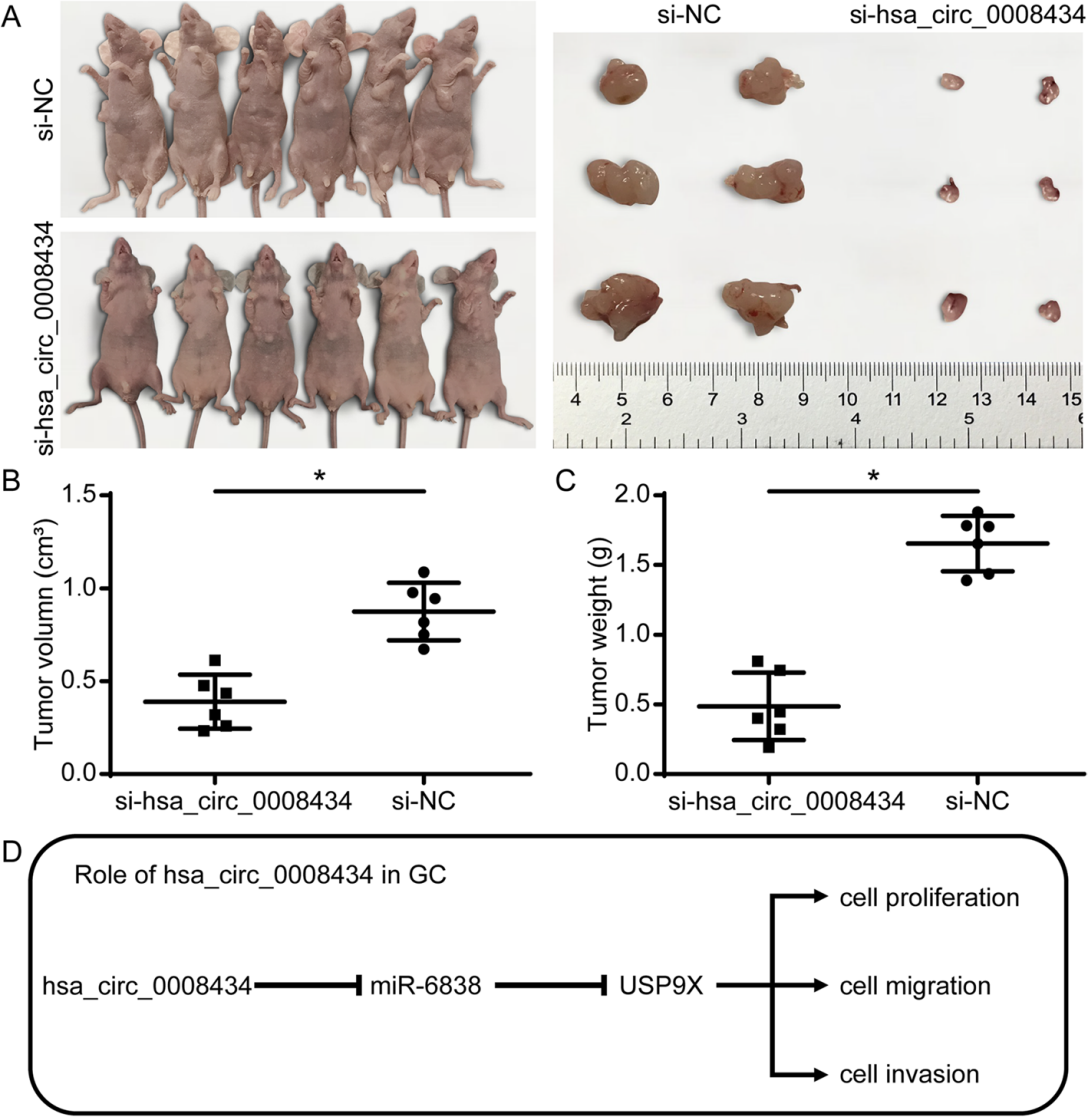
Hsa_circ_0008434 promotes the growth ability of tumours in vivo. Subcutaneous injection of SGC7901 cells transfected with si-hsa_circ_0008434 or si-NC into nude mice was performed to establish flank xenograft models (three mice per group). **A** The growth ability of tumours was evidently decreased in the si-hsa_circ_0008434 group. The tumour volume (**B**) and weight (**C**) were compared between the two groups. **D** The functional role of hsa_circ_0008434 in GC. ^*^p < 0.05

To investigate the role of hsa_circ_0008434 in GC cell growth in vivo, we established a xenograft tumour model in nude mice to observe tumour growth after subcutaneous injection with SGC7901 cells. Tumour growth was decreased significantly in the hsa_circ_0008434 knockdown group compared with the si-NC group (Fig. 5A). In addition, the tumour volume (Fig. 5B) and weight (Fig. 5C) were significantly lower in the hsa_circ_0008434 knockdown group than in the si-NC group. The above results indicated that hsa_circ_0008434 could promote tumour growth in vivo.

## Discussion

In recent years, an increasing number of studies have shown that circular RNAs are closely related to the development of various human malignant tumours [19, 20]. An increasing number of circRNAs have been found to play an important role in many tumours by acting as miRNA sponges, protein decoys, and translation regulators [21]. Recent studies have shown that circ-RanGAP1 [22] and circDUSP16 [23] are upregulated in GC and have an important role in GC proliferation, invasion, and metastasis. Another study showed that circRNA_001569 could promote the proliferation and inhibit the apoptosis of GC cells via the miR-145/NR4A2 axis [24]. CircRNA_0023642 was shown to serve as a metastasis activator by promoting epithelial-mesenchymal transition (EMT) in GC [25]. In contrast, Wang and colleagues [26] found a significant downregulation of hsa_circ_0027599 in GC tissues and cells, as well as a negative relationship between circ_0027599 expression and tumour size and the tumour tumour-node-metastasis (TNM) stage. Zhang et al. found that circLARP4 could inhibit the proliferation and invasion of GC cells [27]. These studies indicate that circRNAs play an important role in the pathogenesis of GC, with both positive and negative effects. In this study, we observed the upregulation of hsa_circ_0008434 in GC tissue for the first time. Moreover, we discovered that hsa_circ_0008434 could promote the proliferation, invasion, and migration of GC cells in vitro and tumour growth in vivo.

CircRNAs can act as miRNA sponges, competing for miRNA binding as competitive endogenous RNAs (ceRNAs), which can negatively regulate miRNA activity [8, 28]. For example, hsa_circ_0092306 can promote GC development by sponging miR-197-3p [29]. CircLMTK2 acts as a sponge of miR-150-5p and promotes GC proliferation and metastasis [30]. CircDLST promotes GC tumorigenesis and metastasis by sponging miR-502-5p [31]. In the present study, we found that hsa_circ_0008434 promotes GC cell proliferation, invasion, and migration by binding to miR-6838-5p.

Further exploration of the downstream genes revealed that USP9X may be regulated by hsa_circ_0008434 and miR-6838-5p. USP9X is a ubiquitin protease that has recently been proven to have an important role in cancer development [14]. A previous study found that USP9X could promote the occurrence of breast cancer by regulating CEp131 [32]. Another study found that USP9X inhibits tumour formation by regulating the stability of the FBW7 protein in colorectal cancer [33]. In some cancers, USP9X has been shown to modulate chemoresistance. Inhibition of ubiquitin activity of USP9X can enhance the cytotoxicity of cisplatin in ER-negative tumour cells [34] and increase the sensitivity of hepatoma cells to adriamycin through USP9X-dependent P53 degradation [35]. Unfortunately, the role and mechanism of USP9X in GC remain unexplored. In the present study, we discovered that hsa_circ_0008434 could enhance the expression of USP9X by sponging miR-6838-5p and further promote USP9X to play a role in promoting GC progression. According to our findings, further molecular mechanism research on USP9X and hsa_circ_0008434 may help improve anti-USP9X therapy.

In our study, although we identified the relationship between hsa_circ_0008434, miR-6838-5p and USP9X and verified the possible biological effects in GC cells (Fig. 5D), we were limited in verifying the deeper mechanism through in vivo experiments. We will carry out in-depth research to supplement and verify the results of this study. It is hoped that our research can identify a direction for future research in GC and find a new target for the treatment of GC as soon as possible.

## Conclusions

Hsa_circ_0008434 regulates the biological behaviour of GC by inhibiting the expression of miR-6838-5p and inducing the expression of USP9X. Hsa_circ_0008434 may be used as a potential biomarker for GC.

## Supplementary Information

**Additional file 1.**

## Data Availability

The datasets used and/or analysed during the current study are available from the corresponding author upon reasonable request.

## Abbreviations

GC: Gastric cancer
circRNAs: Circular RNAs
RBPs: RNA-binding proteins
USP9X: Ubiquitin-specific peptidase 9X
miR-6838: microRNA-6838-5p
FBS: Fetal bovine serum
TPM: Transcript per million
PCA: Principal component analysis
GO: Gene Ontology
KEGG: Kyoto Encyclopedia of Genes and Genomes
UTRs: Untranslated regions
RIPA: Radioimmunoprecipitation assay lysis buffer
DAPI: 4,6-diamidino-2-phenylindole
ANOVA: Analysis of variance
RNA-seq: RNA sequencing
ceRNAs: Competitive endogenous RNAs.
